# An Innovative Synbiotic Formulation Decreases Free Serum Indoxyl Sulfate, Small Intestine Permeability and Ameliorates Gastrointestinal Symptoms in a Randomized Pilot Trial in Stage IIIb-IV CKD Patients

**DOI:** 10.3390/toxins13050334

**Published:** 2021-05-05

**Authors:** Carmela Cosola, Maria Teresa Rocchetti, Ighli di Bari, Paola Maria Acquaviva, Valentina Maranzano, Simone Corciulo, Agostino Di Ciaula, Domenica Maria Di Palo, Flavia Maria La Forgia, Sergio Fontana, Maria De Angelis, Piero Portincasa, Loreto Gesualdo

**Affiliations:** 1 Nephrology, Dialysis and Transplantation Unit, Department of Emergency and Organ Transplantation, University of Bari “Aldo Moro”, 70124 Bari, Italy; carmela.cosola@uniba.it (C.C.); ighli.dibari.uniba@gmail.com (I.d.B.); acquaviva.paola@yahoo.it (P.M.A.); valentia301@gmail.com (V.M.); simo.corciulo@gmail.com (S.C.); 2 Department of Clinical and Experimental Medicine, University of Foggia, 71122 Foggia, Italy; mariateresarocchetti70@gmail.com; 3 Department of Biomedical Sciences and Human Oncology, Clinica Medica “A. Murri”, Medical School, University of Bari “Aldo Moro”, 70124 Bari, Italy; agostinodiciaula@tiscali.it (A.D.C.); domenicamariadipalo@gmail.com (D.M.D.P.); piero.portincasa@uniba.it (P.P.); 4 Centro Studi e Ricerche Dr. Sergio Fontana (1900–1982), 76012 Canosa, Italy; f.laforgia@farmalabor.it (F.M.L.F.); sergiofontana@farmalabor.it (S.F.); 5 Department of Soil, Plant and Food Science, University of Bari “Aldo Moro”, 70126 Bari, Italy; maria.deangelis@uniba.it

**Keywords:** chronic kidney disease, uremic toxins, intestinal permeability, indoxyl sulfate, randomized pilot trial, synbiotic, gut microbiota

## Abstract

Proteolytic dysbiosis of the gut microbiota has been recognized as both a typical feature of chronic kidney disease (CKD) and a risk factor for its progression. Blood accumulation of gut-derived uremic toxins (UTs) like indoxyl sulfate (IS) and p-cresyl sulfate (PCS), intestinal permeability and constipation are typical features accompanying CKD progression and triggering chronic inflammation. In order to verify the efficacy of the innovative synbiotic formulation NATUREN G^®^ in modulating the levels of circulating UTs, intestinal permeability and gastrointestinal symptoms, we set up a randomized, single-blind, placebo-controlled, pilot trial in stage IIIb-IV CKD patients and in healthy controls. Two-month administration of the synbiotic resulted in a decrease of free IS, as compared with the placebo-treated arm, only in the CKD group. The other UTs did not significantly change, although different trends in time (increase in the placebo arm and decrease in the synbiotic arm) were observed. Moreover, after supplementation, reduction of small intestinal permeability and amelioration of abdominal pain and constipation syndromes were observed only in the CKD group. The obtained results suggest the specificity of action of NATUREN G^®^ in CKD and justify further validation in a wider study population.

## 1. Introduction

The importance of gastrointestinal microbiome status in chronic kidney disease (CKD) is an emerging topic in literature, progressively recognized in the last years as both a typical feature of the disease itself and a risk factor for its progression [1,2]. Indeed, CKD patients display a progressive worsening of proteolytic dysbiosis of the gut microbiota, accompanying the progression of the pathology towards end-stage renal disease [1,3]. At the colonic level, the release of nitrogen metabolites in compensation for the compromised renal excretory function exerts selective pressure on microbial populations, favoring the proliferation of urease and uricase positive species, thus orienting the microbiota composition and metabolism towards a proteolytic profile [4]. This is responsible for the increased production at the colonic level of microbiota-derived proteolytic uremic toxins, such as indoxyl sulfate (IS), p-cresyl sulfate (PCS), trimethylamine-N-oxide (TMAO) and indoleacetic acid (IAA) [4,5]. The constipation status frequently observed in these patients further contributes to the proteolytic dysbiosis due to the consumption of saccharolytic fermentation substrates [6], representing a risk for CKD progression [7]. In parallel, a derangement of the integrity of the gastrointestinal barrier has been reported with a leaky gut syndrome that contributes to trigger a local and systemic low-grade inflammatory status by exposing microbial and food antigens to the underlying gut-associated lymphoid tissue and allowing the passage in the circulation of microbial products [8,9,10]. The increased production of uremic toxins (UTs) at the colonic level, together with their reduced renal clearance and their facilitated passage to the systemic circulation, thanks to the altered mucosal integrity, contribute to their progressive accumulation in blood as the disease progresses overall. Their documented actions in promoting inflammation, oxidative stress, insulin resistance and cardiovascular risk makes them active contributor of CKD progression [1,2,5]. Given this strict relationship between gut and kidney functionality, an active field of research is focusing on different strategies of gut microbiota modulation by nutrition and supplementation approaches, with the final aim of blunting CKD progression. The rationale of these strategies mainly stands on the hypothesis that effective manipulation of gut microbiota balance towards a saccharolytic profile would indeed result in the decrease of UTs production and an increase of the anti-inflammatory and barrier-cementing short-chain fatty acids (SCFAs) [11,12]. This, in turn, would reduce UTs inflammatory and oxidant action, both systemically and especially at the renal and cardiovascular levels, thus slowing down CKD progression. In parallel, also increasing the efficacy of UTs dialytic removal represents another area of active investigation aimed at limiting the cardiovascular risk in hemodialysis (HD) patients. In this regard, our group recently demonstrated the effectiveness of a combined approach between synbiotic treatment and innovative dialytic strategies in reducing both IS and PCS serum levels [13]. Currently, there is weak evidence that such approaches could delay the progression of CKD [14,15], and the need is felt to increase the bulk of evidence in this regard.

To this purpose, we set up a randomized, single-blind, placebo-controlled, pilot trial in stage IIIb-IV CKD patients and healthy controls to study the efficacy of an innovative synbiotic formulation in modulating the serum levels of microbiota-derived uremic toxins. Moreover, we aimed to investigate the effects of the synbiotic on the barrier permeability of different gastrointestinal tracts and on gastrointestinal symptoms assessed by the GSRS questionnaire. 

## 2. Results

### 2.1. Clinical Trial, Baseline Characteristics and Clinical Results

**Figure 1:**
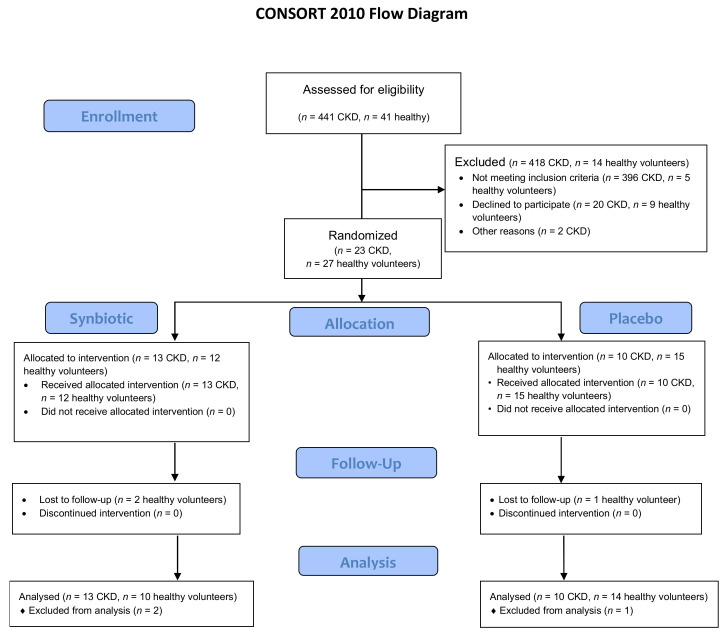
CONSORT study design. Eligibility was assessed in 441 CKD patients, and 41 healthy volunteers with 23 CKD patients and 27 healthy volunteers enrolled and randomized in the Synbiotic (S) (*n* = 13 CKD, *n* = 12 healthy) and Placebo (P) arm (*n* = 10 CKD, *n* = 15 healthy). No CKD patient dropped out, while in the healthy volunteer group, three subjects dropped out, two allocated in the S-arm and one in the P-arm.

In this clinical trial, two groups of 23 CKD stage IIIb-IV patients and 27 healthy volunteers were enrolled and underwent single-blinded randomization for either receiving the synbiotic NATUREN G^®^ (arm S, *n* = 13 CKD, *n* = 12 healthy volunteers) or a placebo (arm P, *n* = 10 CKD, *n* = 15 healthy volunteers). The randomization was performed by an independent researcher by means of software, using gender as a blocking factor. Patients were enrolled and examined by a physician, while questionnaires (gastrointestinal symptoms rating scale (GSRS), Bristol, food frequency questionnaire (FFQ)) were administered by a trained dietitian. The powder formulations were anonymously labelled and were undistinguishable for the patients. All the CKD patients completed the study. In the group of healthy volunteers, three persons dropped out, two belonging to the S-arm and one to the P-arm (Figure 1).

Table 1 and Table 2 show baseline characteristics of the enrolled CKD and healthy populations, respectively. In the CKD cohort, after randomization, no difference was detected for all the clinical parameters examined, with the exception of serum creatinine, significantly lower in the S-arm (S 2.3 ± 0.8 vs. P 3.2 ± 1.1 mg/dL, *p* = 0.04). Moreover, the estimated glomerular filtration rate (eGFR) did not significantly differ between the arms (Table 1). In the healthy volunteer group, no difference was observed in clinical parameters, with the exception of diastolic blood pressure (S 78.3 ± 6.6 vs. P 68.8 ± 7.4 mmHg, *p* = 0.01) and serum triglycerides (S 91 ± 29.4 vs. P 65.8 ± 24.5 mg/dL, *p* = 0.04), significantly higher in the S-arm (Table 2).

Table 3 and Table 4 show the changes in clinical parameters after the treatment with either the placebo or the synbiotic (T2) and after the washout (T3) in the CKD and in the healthy volunteer populations, respectively. In the CKD group (Table 3), no significant differences were observed between the arms, except for the serum sodium, which slightly and significantly increased at T2 in the S-arm. The baseline difference in serum creatinine was maintained between the arms, also at T2 and T3. At the end of the trial, the patients treated with the synbiotic showed a significant decrease in azotemia (T3 87.08 ± 22.3 vs. T2 94.08 ± 25.17 mg/dL, *p* = 0.01) and calcium-phosphorus product (T3 23.72 ± 11.54 vs. T2 31.81 ± 3.90 mg^2^/dL^2^, *p* = 0.03) as compared to T2. In the P-arm, changes in the serum levels of electrolytes were observed at different time points, with a significant decrease of serum sodium, while sodium levels increased at T2 in the S-arm in comparison to baseline. The latter changes seem more attributable to physiological fluctuations of the electrolyte levels than to the treatment with the synbiotic or placebo. CRP and IL6 were unchanged after the synbiotic supplementation (Table 3, Supplementary Figure S1), indicating no specific effects of NATUREN G^®^ on inflammatory and oxidative parameters. Systolic and diastolic blood pressure and metabolic parameters did not change significantly between different time points or between arms.

In the healthy group (Table 4), no significant differences were present between the arms at the different time points after the treatment, except for the diastolic blood pressure (T2, S 75 ± 8.3 vs. P 68.1 ± 5.2 mmHg, *p* = 0.03; T3, S 77.8 ± 10.6 S vs. P 68.1 ± 8.1 mmHg, *p* = 0.03) and the triglycerides level (T2, S 95.6 ± 30.8 vs. P 66.5 ± 24.1 mg/dL, *p* = 0.02; T3, S 114.6 ± 43 vs. P 75.9 ± 25.6 mg/dL, *p* = 0.02), whose baseline differences between the arms remained unchanged across the different time points.

### 2.2. NATUREN G^®^ Reduces Serum Concentration of Free IS Only in CKD Patients

**Figure 2:**
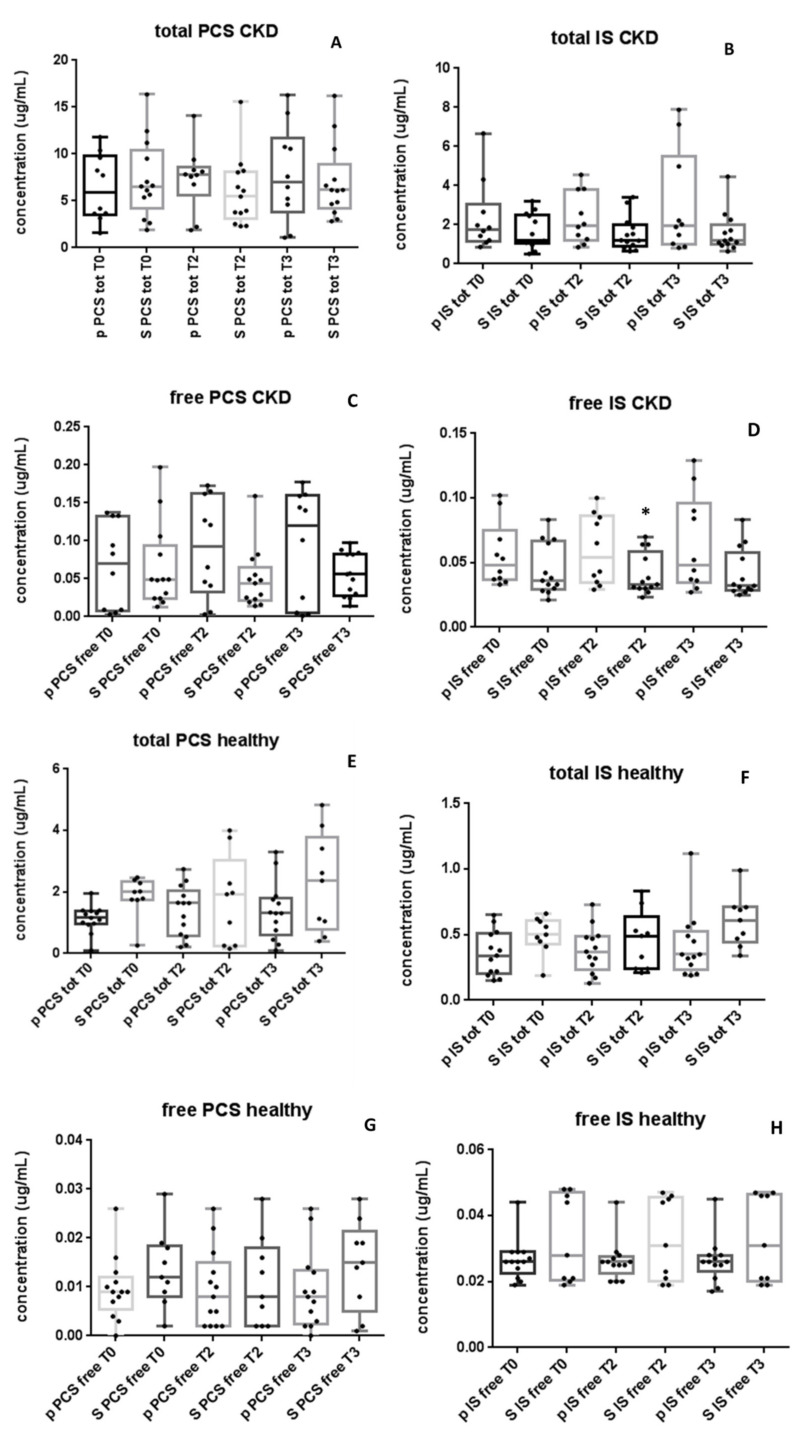
NATUREN G^®^ reduces serum concentration of free IS only in the CKD group. The figure shows the serum levels of total PCS (**A**) and IS (**B**) and free PCS (**C**) and IS (**D**) in the CKD group (13 S and 10 P). (**E**–**H**) show the serum levels of total PCS, total IS, free PCS and free IS in the healthy volunteer group (10 S and 14 P). Only in the CKD group, at T2, free IS levels were significantly lower in the S-arm as compared with the P-arm (**D**). * *p* < 0.05 vs. placebo. Data are expressed with medians, interquartile ranges and minimum to maximum values, differences tested by Kruskal–Wallis multiple-comparison *z*-value test.

At baseline, average concentrations of PCS and IS, both total and free forms, did not significantly differ between S and P-arms, either in the CKD and in the healthy volunteer groups, indicating homogeneous randomization (Figure 2A).

In the CKD group, after two months of either NATUREN G^®^ or placebo administration, a significant reduction of free IS in the S-arm as compared with the P-arm (S 0.033 [0.030, 0.046] vs. P 0.054 [0.034, 0.086] µg/mL, *p* = 0.02) was observed (Figure 2D). The difference of free IS between S- and P-arms was also maintained after the washout period (S 0.032 [0.029, 0.058] vs. P 0.048 [0.035, 0.096] µg/mL) but without statistical significance. Total IS and PCS (Figure 2A,B) and free PCS (Figure 2C) did not show significant variations between arms after the treatment nor after the washout period, even though a trend towards their progressive increase in time can be observed only in the P-arm. On the contrary, in the S-arm, a trend of reduction of the total IS and PCS and free PCS was observed after synbiotic treatment (between T0 and T2; Figure 2A–C).

In the healthy volunteer group, treatment with either NATUREN G^®^ or placebo did not result in significant differences in PCS or IS, either in total or free forms (Figure 2E–H).

### 2.3. Whole Gut Permeability in Healthy Subjects and in CKD Patients

Gastrointestinal permeability was evaluated at baseline on 22 CKD patients (one patient belonging to the P-arm did not perform the test for personal reasons) and in the group of 27 healthy subjects (Table 5).

**Figure 3:**
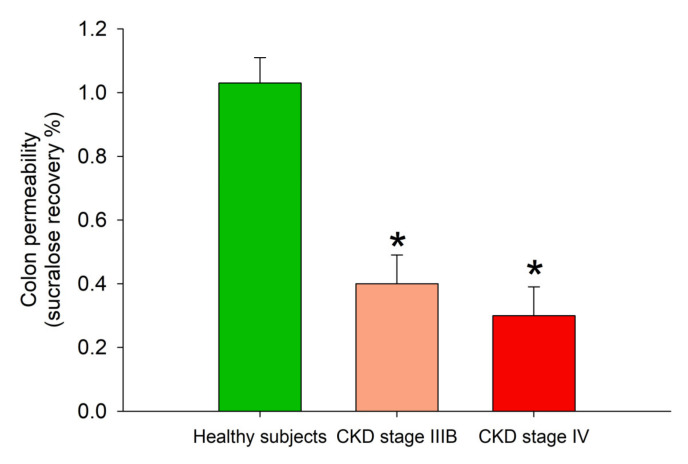
Baseline colon permeability, as measured by % of sucralose recovery, in healthy subjects and in CKD patients in stage IIIB and IV. * *p* < 0.05 vs. healthy subjects (one-way ANOVA followed by Fisher’s LSD Multiple comparison test).

At baseline, CKD patients and healthy subjects showed comparable gastric and small intestinal permeability. Sucralose recovery (expression of colon permeability), however, was decreased in CKD (Table 5, Figure 3).

**Figure 4:**
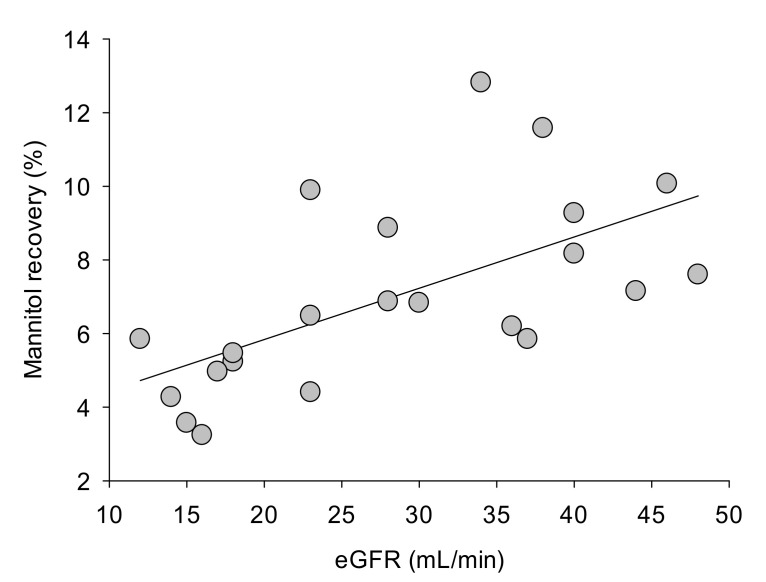
Linear regression analysis between mannitol recovery (%) and eGFR (mL/min) in CKD patients. Measurements were taken at entry (baseline). R = 0.60. *p* = 0.0034.

**Figure 5:**
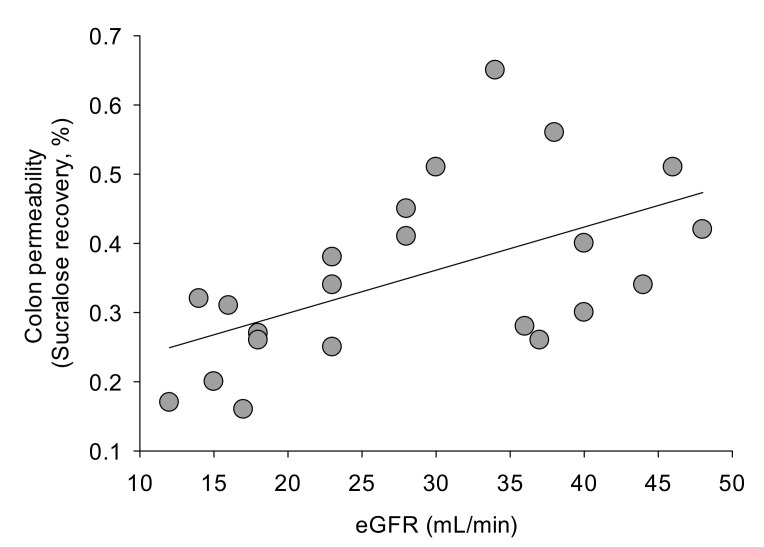
Linear regression analysis between sucralose recovery (%), an expression of colon permeability and eGFR (mL/min) in CKD patients. Measurements were taken at entry (baseline). R = 0.54. *p* = 0.01.

A negative correlation between age and small intestine permeability was detected in CKD patients (R = −0.45, *p* = 0.03), but not in healthy subjects. Age did not correlate with gastric or colon permeability in healthy subjects or in CKD patients (data not shown). No difference in terms of gastrointestinal permeability was evident in healthy subjects or in CKD patients when subjects were stratified according to body mass index (BMI) (i.e., normal weight or overweight subjects, data not shown). In CKD patients, the recovery of mannitol (Figure 4) and sucralose (Figure 5) were positively correlated with the eGFR. This was not the case, however, for sucrose and lactulose recovery.

The effects of placebo or synbiotic administration on permeability at different levels in the gastrointestinal tract were evaluated in a final group of 21 CKD patients (an additional patient belonging to the S-arm did not complete the procedure), and in all the 24 healthy subjects that completed the study. The results are reported in Table 6 (CKD patients) and in Table 7 (healthy subjects). As expected, the administration of placebo did not modify gastric, small intestine and colon permeability neither in CKD patients (Table 6) nor in healthy subjects (Table 7), with comparable measurements at baseline, after supplementation (T2), and after washout (T3). In CKD patients (Table 6), small intestinal permeability decreased significantly after 2 months of treatment (T2), remaining stable after the washout period (T3). Synbiotic administration did not change stomach and colon permeability. The treatment with synbiotic did not influence gastrointestinal permeability (all levels) in healthy subjects (Table 7).

### 2.4. NATUREN G^®^ Ameliorates Gastrointestinal Symptoms in the CKD Group

Gastrointestinal symptoms were evaluated by GSRS questionnaire, both in CKD and in the healthy group. In the CKD group, at baseline, no significant difference was observed between the P- and S-arms in any of the 15 items. After treatment, we observed a significant decrease of the scores related to items 6 (rumbling) and 13 (hard stools) only in the arm treated with NATUREN G^®^ (Table 8) as compared to baseline, although these scores were not significantly different with those of the P-arm at T2 and T3. In particular, after the two-month synbiotic administration, hard stool (T2 1.15 ± 0.15 vs. T0 2.23 ± 0.27, *p* = 0.02) item ameliorated in the S-arm, without significant changes in the P-arm. After the washout, the hard stool score remained significantly lower than baseline (T3 1.31 ± 0.20 vs. T0 2.23 ± 0.27, *p* = 0.03), while rumbling score became significantly lower than T0 (T3 1.46 ± 0.23 vs. T0 2.38 ± 0.34, *p* = 0.04). In parallel, after the two-month treatment with NATUREN G^®^, the overall domains of abdominal pain and constipation syndrome improved significantly in the S-arm in comparison with the beginning of the study (abdominal pain T2 3.54 ± 0.28 vs. T0 4.85 ± 0.51, *p* = 0.03; constipation syndrome T2 3.77 ± 0.41 vs. T0 6.08 ± 0.80, *p* = 0.02). No differences existed between the different time points for the remaining scores and in no score in the P-arm.

In the healthy group, at baseline, no significant differences were observed between the P- and S-arms in any of the 15 items. Differences were observed in items 3 (acid regurgitation) and 9 (increased flatus) and the indigestion syndrome in the P-arm at T2 vs. baseline, probably ascribable to other factors independent from the synbiotic administration. Amelioration of the abdominal pain domain was observed in P-arm, either at T2 and at T3. No differences were detected between the different time points for the remaining scores and domains (Table 9).

### 2.5. Total and Free IS Directly Correlate with Azotemia in Synbiotic-Treated CKD Patients

**Figure 6:**
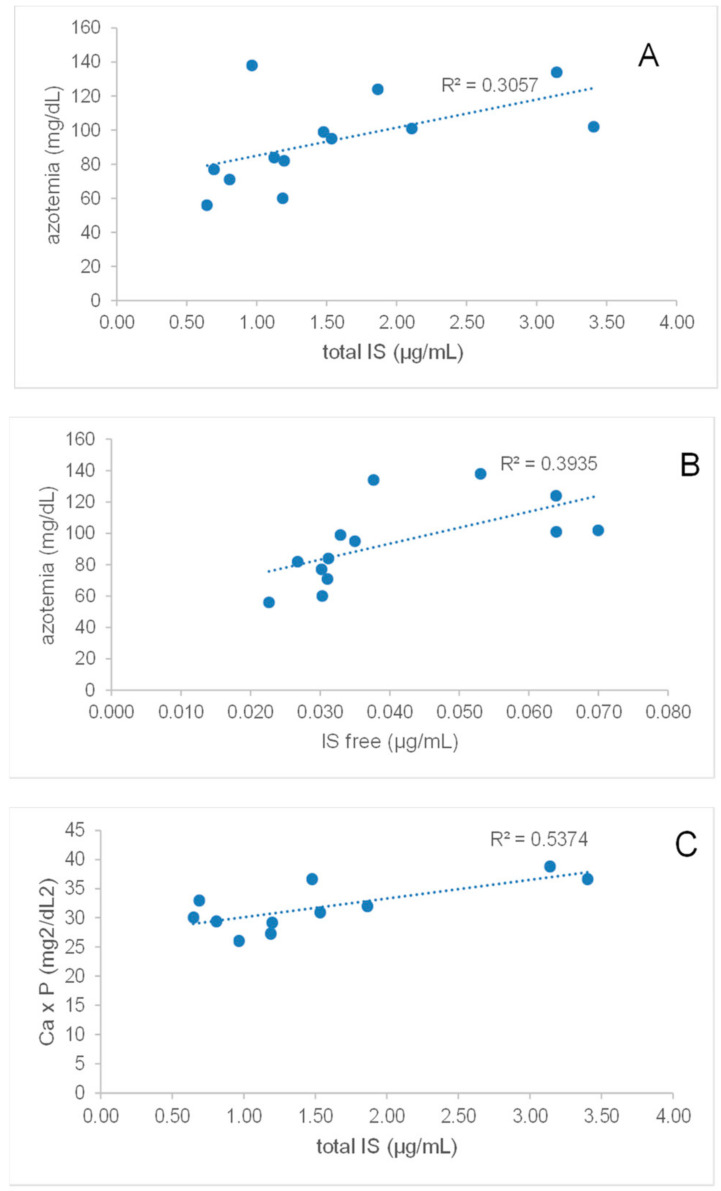
Spearman correlations between azotemia and total IS (**A**) or free IS (**B**); correlation of Ca × P product with total IS (**C**).

After supplementation with NATUREN G^®^, only in patients belonging to the S-arm (*n* = 13), we observed positive and significant correlations between IS and azotemia and calcium-phosphorus product after the treatment (Figure 6). Indeed, azotemia directly correlated with total IS (ρ = 0.66, *p* = 0.02) and free IS (ρ = 0.85, *p* = 0.0004), while a significant correlation with both forms of PCS was lacking. Moreover, total IS showed a trend of correlation with the calcium-phosphorus product, although not statistically significant (ρ = 0.54, *p* = 0.09).

## 3. Discussion

In this single-blind, randomized, controlled pilot trial, we demonstrate that a two-month treatment with the synbiotic NATUREN G^®^ is effective in reducing serum free IS, small intestine permeability, abdominal pain and constipation syndromes in stage IIIb-IV CKD patients. This action is selective for CKD since changes in UTs serum levels, gastrointestinal permeability and gastrointestinal symptoms are not observed in healthy volunteers treated with the same synbiotic for the same time interval.

It is well established that an alteration of the gut microbiota accompanies CKD and likely contributes to its progression and cardiovascular and inflammatory complications [1,2]. The CKD-associated dysbiosis is characterized by a prevalence of proteolytic species, with increased production and reduced clearance of uremic toxins, such as IS, PCS, TMAO and IAA [4,5]. Constipation and intestinal permeability are two other typical CKD-associated factors [16], respectively representing a risk for CKD progression [7] and triggering local and systemic inflammation [8]. The dynamics of the above-described gut-kidney axis led to the development of a field of research aiming to manipulate gut microbiota in order to reduce UTs production, alleviate inflammatory and oxidative outcomes, and delay CKD progression [12,15].

In particular, the present work finds its place in a current area of research comprising a group of heterogeneous trials, which have been carried on in CKD patients at different stages, in order to investigate whether supplementation with probiotics, prebiotics or synbiotics could be effective in lowering UTs. Conflicting results are reported in literature following supplementation with fructooligosaccharides (FOS) in CKD patients at intermediate stages. Indeed, a study reported the efficacy of these prebiotics in reducing both total and free-circulating PCS, without changing IS and IAA levels nor gastrointestinal symptoms or markers of intestinal permeability [17], while in another study, FOS showed effects on inflammation and endothelial function, but only a trend towards PCS reduction [18]. Another crossover trial on CKD patients showed no effects of a 4-week supplementation with prebiotic arabinoxylan oligosaccharides on UTs, except for a slight reduction in TMAO [19]. Similar to the present study, synbiotic treatments seem to display the most promising results, suggesting synergic action of both the probiotic and prebiotic components in CKD patients. A mixture of different strains of Lactobacilli, Bifidobacteria, Streptococcus, FOS, inulin and resistant starch was administered for 4 weeks to stage 3-4 CKD patients, resulting in total PCS reduction but in no amelioration of gastrointestinal (GI) symptoms [20]. Another trial on CKD patients explored the effects of a synbiotic composed of inulin, FOS, galactooligosaccharides (GOS) and different strains across the Lactobacillus, Bifidobacteria and Streptococcus genera, observing a reduction in PCS but not IS, nor an amelioration of GI symptoms [21].

In this trial, we administered the innovative synbiotic NATUREN G^®^, composed of a mixture of *Lactobacilli* and *Bifidobacteria* species, FOS and inulin as prebiotics, and natural antioxidants, or a placebo formulation for two months to stage IIIb-IV CKD and healthy populations. Only in the synbiotic-treated CKD arm, we observed a significant reduction in serum free IS. This difference was sustained also after a one-month washout period, though losing statistical significance, indicating a positive drag effect on microbiota alteration. It is worthy to observe that the free forms of UTs should be considered the real target of these strategies since UTs mainly circulate bound to albumin, being the free fractions the biologically active forms. The other analyzed UTs, PCS (both total and free form) and total IS similarly showed a trend towards reduction after the treatment with NATUREN G^®^, without reaching statistical significance. It is interesting to notice that in the placebo-treated CKD arm, a trend towards UTs progressive increase in time can be observed, and this increase seems to be blunted by the synbiotic administration. The described effects were only observed in the CKD population and not in healthy volunteers, suggesting the specificity of action of the novel synbiotic. No differences were observed in the analyzed clinical parameters, both in CKD and in the healthy population, except for a slight but significant reduction of azotemia and Ca × P product in the S-arm of the CKD group observed after the washout. The clinical significance of this observation is unknown; however, one hypothesis that could explain this observation is a new balance of gut microbiota population, established after discontinuing the synbiotic supplementation. A reduced ammonia intestinal availability because of reduced proteolytic species would indeed result in a reduced substrate for the hepatic urea cycle [4]. Moreover, as previously observed by our group in a study on CKD patients [22], we confirm here that azotemia directly and significantly correlates with both total and free IS. Differently, in spite of the enrichment of the synbiotic with quercetin, resveratrol and proanthocyanidins, we failed to observe a specific action of these components on inflammatory/oxidative parameters. This could depend on the moderate baseline inflammatory status in the studied cohort, as evidenced by CRP levels in the normal range. Alternatively, the nutraceutical supply in the form of a supplement rather than food could have hampered their effect [23,24].

It is established that in CKD, like in other dysbiotic conditions, alterations of the integrity of the gastrointestinal barrier do exist [8]. Bacterial products have been detected in the blood of end-stage renal disease (ESRD) patients and have been reported to correlate with the inflammatory status [25,26], while in a CKD animal model intestinal permeability and its associated aberrant mucosal immunity have been linked to kidney fibrosis [9]. Our group previously demonstrated a reduction of intestinal permeability in a cohort of CKD patients, following dietary manipulation, in parallel with the reduction of azotemia, IS and PCS serum levels. Interestingly, intestinal permeability assessed by serum D-lactate and azotemia directly correlated [22]. In this study, we demonstrate, for the first time in CKD patients in our knowledge, that the synbiotic formulation tested here is able to slightly but significantly reduce small intestinal permeability, as demonstrated by the urinary recovery of lactulose and mannitol. The decrease of this marker of the intestinal barrier remains even after a one-month washout. Differently from lactulose and mannitol, CKD patients show decreased sucralose recovery, an expression of colon permeability. The altered sucralose recovery in patients with CKD was not expected. However, a possible influence of an altered renal function on urinary recovery of the four oligosaccharide probes following simultaneous oral administration was a possible event. Thus, we checked the existence of possible correlations between individual eGFR and urinary recovery of mannitol, sucralose, sucrose and lactulose. We found a positive correlation between the recovery of mannitol and sucralose and the eGFR values. This was not the case for sucrose and lactulose recovery. This evidence should support the hypothesis of a decreased sucralose recovery as an expression of altered renal function, rather than of abnormal colon permeability. However, the existence of an ongoing abnormal colonic permeability to sucralose cannot be fully excluded. Colonic permeability, in fact, is partly dependent on epigenetic mechanisms, and a greater expression of some miRNAs has been reported in subjects with normal kidney function, as compared with CKD [27]. Among these, miR-21 is significant related with eGFR [27]. In animal models, miR-21 knockdown significantly decreased intestinal permeability, acting on intestinal epithelial tight junctions [28]. Furthermore, miR-29a levels are significantly lower in CKD patients as compared with controls [29]. Of note, animal [30] and human studies [31] have documented a direct relationship between miR-29a levels and the extent of intestinal permeability. Taken together, and besides the effects of impaired renal excretion of sucralose per se, these findings could explain, at least in part, the decreased colon permeability of sucralose observed in the present cohort of CKD patients. Further studies, however, are needed to better assess the translational relevance of these findings in patients with CKD. In addition, following synbiotic administration, we found a significant improvement of gut permeability at the level of the small intestine. This intestinal segment is significantly less colonized by microbes than the colon in healthy subjects. However, the small intestine (i.e., duodenum, jejunum) can become heavily colonized in CKD patients in terms of both anaerobes and aerobes [32]. Furthermore, small intestinal bacterial overgrowth, as well as abnormal motility, are common in patients with chronic renal failure [33]. On the other hand, small intestinal bacterial overgrowth has been linked with abnormal small intestinal permeability [34]. Thus, in the present group of CKD patients, positive changes in microbiota composition following treatment with the synbiotic NATUREN G^®^ might have generated a global improvement in small intestine permeability. This result, however, should be confirmed by further observations in a larger number of subjects. We previously reported that colonic permeability increased in patients with nonalcoholic liver steatosis or low adherence to the Mediterranean diet [35]. Similar to the other outcomes, healthy subjects undergoing NATUREN G^®^ supplementation do not show any difference in gastrointestinal permeability after the treatment, suggesting again a selective action of the synbiotic in the CKD setting.

We also demonstrate here that the two-month supplementation with NATUREN G^®^ exerts beneficial effects on GI symptoms by ameliorating abdominal pain and constipation syndrome only in synbiotic-treated CKD patients, with no significant effects in the healthy group. Given the focal importance of treating constipation in CKD patients [6], the amelioration of the item related to the hardness of stools, which we observed in the present study at the end of NATUREN G^®^ supplementation and was maintained significantly better even after the washout, acquires particular importance in this setting. It should also be considered that other similar studies carried on with synbiotics administered to CKD patients at intermediate stages failed to observe ameliorations in gastrointestinal symptoms [17,20,21].

In conclusion, this pilot trial demonstrates the selective efficacy, only in CKD patients and not in non-CKD subjects, of NATUREN G^®^ in reducing free circulating IS and in improving small intestine barrier integrity, both at the end of the supplementation and after one-month washout, in parallel with a reduction of azotemia. Moreover, NATUREN G^®^ supplementation ameliorates both abdominal pain and constipation syndromes, and the latter was maintained after the washout.

The main limitation of our work is the limited sample size due to the exploratory nature of this pilot trial aimed to investigate the effects of the novel synbiotic NATUREN G^®^. Moreover, in this trial, patients with type 2 diabetes mellitus were excluded, and this represents an additional limitation to the generalizability of this study, given the large proportion of diabetic patients amongst the CKD population. The strengths of the work stand in the duration of the study (two months instead of four weeks reported by the majority of studies) and in the study design, allowing us to compare the effects of the synbiotic treatment to a placebo, both in the CKD population and in a cohort of healthy subjects, and also to observe the drag effects after one-month synbiotic discontinuation. The obtained results suggest the safety and potential usefulness of periodic cycles of supplementation in CKD patients and certainly justify the experimentation of NATUREN G^®^ supplementation on a wider CKD population at intermediate stages.

## 4. Materials and Methods

### 4.1. Study Design, Clinical Parameters and GSRS/Bristol Scores

The goal of this randomized, single-blind, placebo-controlled, pilot trial in CKD patients and healthy controls was to validate the efficacy of an innovative synbiotic in modulating the serum levels of microbiota-derived uremic toxins and intestinal permeability.

The trial was carried out according to the Helsinki Declaration (IV Adaptation) and the European Guidelines for Good Clinical Practice. The study started in May 2017 and ended in October 2017. Being a pilot study, no sample size calculation was adopted, establishing a target of 12 participants for each group [36]. The protocol of the study was approved by the Ethical Committee of the Azienda Ospedaliero-Universitaria Consorziale Policlinico of Bari, Italy (Authorization nr. 0673/2017) and registered in ClinicalTrials.gov registry database (Identifier nr. NCT03815786). The authors confirm that all ongoing and related trials for this intervention are registered in the ClinicalTrials.gov registry database; because of administrative issues, the trial was registered after the enrolment of the participants. Participants in the study were enrolled according to the following inclusion/exclusion criteria. CKD population inclusion criteria: CKD patients in stage IIIb-IV not on dialysis, aged between 30 to 65, BMI between 18.5 and 29.9, controlled diet, written informed consent signed. CKD population exclusion criteria: type 2 diabetes mellitus, use of antibiotics or probiotics up to 30 days prior to recruitment, chronic gastrointestinal disorders, systemic inflammatory diseases, suspicion or clinical diagnosis of malignancy, chronic liver disease, treatment with corticosteroids or immunosuppressive drugs, previous acute cardiovascular diseases (myocardial infarction, stroke), psychiatric conditions reducing the compliance to treatment protocols. Healthy population inclusion criteria: age between 35 to 60, BMI between 18.5 and 29.9, a medium score of adherence to the Mediterranean Diet (PREDIMED score [37] between 6 and 9), written informed consent signed. Healthy population exclusion criteria: type 2 diabetes mellitus, use of antibiotics or probiotics up to 30 days prior to recruitment, chronic gastrointestinal disorders, systemic inflammatory diseases, suspicion or clinical diagnosis of malignancy, chronic liver disease, treatment with corticosteroids or immunosuppressive drugs, previous acute cardiovascular diseases (myocardial infarction, stroke) and psychiatric conditions reducing the compliance to treatment protocols. After signing the informed consent, participants of both populations (CKD and healthy volunteers) were randomized for the administration of 2 bags/day of the synbiotic NATUREN G (^®^ Farmalabor SRL) or placebo for 2 months. Patients were blinded for the use of the synbiotic/placebo, which were anonymously and externally indistinguishable. NATUREN G^®^ was composed of a mixture of probiotics (*Lactobacillus Casei* LC4P1 2.4 × 10^9^, *Bifidobacterium Animalis* BLC1 2.4 × 10^9^), prebiotics (fructoligosaccharides 2.5 g and inulin 2.5 g) and natural antioxidants (a mix of quercetin 0.064 g, resveratrol 0.023 g and proanthocyanidins 0.013 g). Primary Outcome: Change of p-cresyl sulfate (pCS) serum concentrations. Secondary Outcomes: Change of serum concentration of IS, change of intestinal permeability, change of serum concentration of inflammatory and oxidative stress markers. Exploratory outcome: change of gastrointestinal symptoms. In this paper, we report clinical and experimental results related to primary and secondary outcomes; results dealing with exploratory outcomes, in particular, regarding gut microbiota composition and changes following the synbiotic administration, will be treated in a separate microbiological paper. TMAO was not evaluated due to technical problems during the execution of the analysis. At the enrolment (T0), after two months of supplementation (T2) and one month of washout (T3), volunteers underwent medical and nutritional examinations, during which the following were collected: blood pressure, blood, urine, FFQ Food Frequency Questionnaire and 24h-recall diary, Gastrointestinal Symptom Rating Scale GSRS. FFQ and 24h-recall were used to monitor compliance of the participants of the study (assumption of the synbiotic/placebo) and to exclude major changes in food habits that could have influenced the outcomes of the trial. Participation to the study was free, and no incentive was provided to the volunteers. Urine and blood samples were processed by the central laboratory of Azienda Ospedaliero-Universitaria Consorziale Policlinico of Bari for routine analyses. Additional aliquots of blood for each patient at each time point of the study were centrifuged at 3000 rpm for 10 min. The obtained serum samples were then stored at −80 °C until use.

Gastrointestinal symptoms were evaluated with the use of a validated questionnaire, the Gastrointestinal Symptom Rating Scale (GSRS) [38]. The GSRS questionnaire is based on a seven-level Likert scale (1–7), according to the intensity and frequency of specific GI symptoms reported by the patient at each study point, with the lowest score indicating a better symptom. The cumulative scores among the questions identify five main domains: “abdominal pain” (pain referred to as epigastric, colic, continuous or indefinite pain, gastric hunger pains and nausea and vomiting: max. score: 21), “reflux syndrome” (heartburn and acid regurgitation: max. score: 14), “indigestion syndrome” (borborygmi, abdominal distension, eructation and increased flatus, max. score: 28), “diarrhea syndrome” (increased frequency of evacuation, loose stools and urgent need to defecate, max. score: 21) and “constipation syndrome” (reduced frequency of evacuation, hard stools and feeling of incomplete evacuation, max. score: 21). The maximal achievable score was 105.

### 4.2. Liquid Chromatography/Electrospray Ionization–Mass Spectrometry/Mass Spectrometry (LC/ESI–MS/MS) for Quantification of pCS and IS

Circulating levels of IS and pCS were determined at the enrollment (T0), after the two-month supplementation with NATUREN G^®^ (T2) and after the one-month washout (T3), for a total of 3 measurements for each patient. Total and free plasma levels of IS and PCS were measured by Multiple Reaction Monitoring (MRM) analysis, using a triple quadrupole mass spectrometer (API4000, AB SCIEX, Carlsbad, CA, USA) equipped with an ESI source and online connected to high-performance liquid chromatography system (CBM-20A LC, Shimadzu, Kyoto, Japan), as previously described by our group [39]. All the samples were run in duplicate to reach an intra-assay coefficient of variation <10%.

### 4.3. Measurement of Gut Permeability

Whole gut permeability was assessed by measuring urinary recovery of four oligosaccharide probes following simultaneous oral administration [40]. The sugars consisted of sucrose (20 g, MW 342.3 g/mol) to measure stomach permeability, lactulose (5 g MW 342.3 g/mol) and mannitol (1 g MW 182.172 g/mol) to measure small intestine permeability, and sucralose (1 g MW 397.64 g/mol) to measure colonic permeability. The procedure followed the guidelines of the manufacturer (AB Analitica s.r.l., Padova, Italy) and experience gained from a recent study from our group [35]. The principle of the test relies on the property of large size molecules to cross the paracellular intestinal pathway only if the intestinal barrier function is compromised. The small size molecule is able to cross the intestinal barrier freely, independent of barrier function loss. For the small intestine, the ratio of the urinary concentration of both molecules (i.e., lactulose and mannitol) measured after 5–6 h appears to reflect more accurately the paracellular passage across the intestinal barrier than isolated measurement of urinary oligosaccharides. Therefore, if the barrier function is lost, the specific probe crosses the intestinal barrier, appears into the circulation and becomes detectable in urines after renal excretion. The permeability process is affected in the same way as the large molecular probe by the pre- and post-mucosal confounders, such as gastric dilution, gastrointestinal motility, bacterial degradation and renal function. Starting one week before the test, subjects were asked to avoid laxatives, prebiotics, probiotics and synthetic sugars. The day before the test, the consumption of chocolate, sweets, chewing gum, fruit juices, fresh or packaged fruit, dietetic products, ice cream and alcohol was inhibited to prevent any potential interference with the test. Subjects could only consume grilled meat or fish and had to drink 1 L of water before bedtime. On the morning of the test, after an overnight fast, a urinary sample was collected before oral administration of the test sugars dissolved in 250 mL of tap water. Urines were, thereafter, collected every hour for six hours in a sterile container containing chlorhexidine. During the whole test, subjects had to remain fasted.

Gastrointestinal permeability was calculated from the 6 h urinary recovery. Two samples for patients were analyzed by urinary plasma chromatography/mass spectrometry (UPLC–MS/MS). The fraction of the excreted sugars was calculated based on the quantity of sugar ingested by the patients and expressed as a percentage value [35,41].

### 4.4. IL6 ELISA

Circulating levels of IL6 were measured by a commercially available Human ELISA Kit colorimetric assay (Thermofisher, Carlsbad, CA, USA) following the manufacturer’s instructions. Briefly, a calibration curve was prepared from serial dilutions following the protocol kit. Biotinylated Antibody Reagent (50 μL), serial dilutions of standards (50 μL) and plasma samples (50 μL) were added in duplicate into a white clear-bottom 96-well microplate. Later, after incubation for 2 h and washing, 100 μL of prepared Streptavidin-HRP Solution was added to each well. The reaction mix was incubated at room temperature for 30 min, protected from light. After washing, TMB solution was added and incubated for 30 min. After adding the stop solution, the absorbance was detected with an absorbance plate reader at A450 nm.

### 4.5. Statistical Analysis and Correlations

The analyses were based on the intention to treat. Experimental data are presented as mean ± SEM, while uremic toxins data are presented as median and interquartile range. Baseline differences between quantitative parametric variables in arms P and S were analyzed by Mann–-Whitney test. Differences between treatments and time points were analyzed by Kruskal–Wallis multiple-comparison *z*-value test followed by Dunn’s post hoc test. Correlations between IS and clinical parameters (azotemia and Ca × P) were performed by the Spearman analysis. Gastrointestinal permeability data are presented as means ± SEM, medians and range. Differences between groups were compared by the Mann-Whitney U test (two groups) or by one-way analysis of variance (ANOVA) followed by Fisher’s LSD multiple-comparison test. Results were considered significant at the 5% critical level (*p* < 0.05). All the analyses were performed using GraphPad Prism (GraphPad software, version 6, San Diego, CA, USA).

## Figures and Tables

**Table 1:** Baseline characteristics of the enrolled CKD population.

	P	S	Difference at Baseline (*p* Value)
Gender	7 M, 3 F	7 M, 6 F	-
Age	51.5 ± 2.8	51 ± 4.3	0.6
BMI	25.8 ± 0.9	27.2 ± 1	0.4
eGFR mL/min	23.9 ± 3.6	31.5 ± 2.8	0.1
Serum creatinine mg/dl	3.2 ± 0.4	2.3 ± 0.2	0.04
sys BP (mmHg)	126.5 ± 3.6	124.2 ± 3.6	0.7
dia BP (mmHg)	82.5 ± 2.5	82.7 ± 2.5	1.0
Serum albumin (g/dL)	3.9 ± 0.1	3.9 ± 0.1	0.8
Azotemia (mg/dL)	97 ± 16	94.9 ± 7.2	0.9
BUN (mg/dL)	45.3 ± 7.5	44.3 ± 3.3	0.9
Blood glucose (mg/dL)	76.4 ± 2.8	78.1 ± 3.4	0.7
HbA1c %	36.8 ± 1.3	35.8 ± 1.1	0.6
Serum tryglicerides (mg/dL)	149.4 ± 22.1	134.7 ± 20.6	0.6
HDL cholesterol (mg/dL)	53.9 ± 3.9	58 ± 5.2	0.6
LDL cholesterol (mg/dL)	101.7 ± 6.6	97.5 ± 10.4	0.8
Total cholesterol mg/dL	174.4 ± 8.4	184.4 ± 11.2	0.5
Serum calcium (mg/dL)	9.2 ± 0.2	9.2 ± 0.1	1.0
Serum phosphorus (mg/dL)	3.4 ± 0.3	3.5 ± 0.1	0.8
Serum potassium (mEq/L)	4.7 ± 0.1	4.5 ± 0.1	0.3
Serum sodium (mEq/L)	141 ± 0.6	142.1 ± 0.6	0.2
CaxP (mg^2^/dL^2^)	31.6 ± 2.7	32.3 ± 1.4	0.8
Total serum proteins (g/dL)	7.2 ± 0.1	7.2 ± 0.1	1.0
CRP (mg/L)	4.1 ± 1.1	3 ± 0.1	0.3
Rrinary creatinine (mg/dL)	66.7 ± 9.5	53.7 ± 3.6	0.2
Proteinuria (mg/dL)	577 ± 211	500.9 ± 117.9	0.8

Data are expressed as mean ± SEM, differences tested by Mann–Whitney test. Abbreviations: BMI (body mass index), eGFR (estimated glomerular filtration rate), BP (blood pressure), HbA1c (glycated hemoglobin), HDL (high-density lipoprotein), LDL (low-density lipoprotein), CaxP (calcium-phosphorus product), CRP (C-reactive protein).

**Table 2:** Baseline characteristics of the enrolled population of healthy volunteers.

	P	S	Difference at Baseline (*p* Value)
Gender	4 M, 10 F	5 M, 5 F	-
Age	42.7 ± 2.7	41.6 ± 3.0	0.8
BMI	23.5 ± 0.8	26 ± 1.6	0.1
Serum creatinine mg/dl	0.79 ± 0.0	0.83 ± 0.0	0.5
sys BP (mmHg)	109.2 ± 2.6	115.6 ± 3.5	0.2
dia BP (mmHg)	68.8 ± 2.1	78.3 ± 2.2	0.01
Serum albumin (g/dL)	4.2 ± 0.1	4.1 ± 0.1	0.4
Azotemia (mg/dL)	32.6 ± 1.1	36.4 ± 3.1	0.2
BUN (mg/dL)	15.3 ± 0.3	17.0 ± 1.4	0.2
Blood glucose (mg/dL)	71.5 ± 2.5	75.2 ± 3.2	0.4
HbA1c %	33.3 ± 1.3	34.6 ± 1.0	0.5
Serum tryglicerides (mg/dL)	65.8 ± 6.8	91 ± 9.8	0.04
HDL cholesterol (mg/dL)	72.7 ± 4.1	72.2 ± 7.4	1.0
LDL cholesterol (mg/dL)	100.2 ± 5.1	113.4 ± 8.5	0.2
Total cholesterol mg/dL	186.8 ± 6.3	204 ± 10.0	0.1
Serum calcium (mg/dL)	9.3 ± 0.1	9.2 ± 0.1	0.1
Serum phosphorus (mg/dL)	3.4 ± 0.1	3.2 ± 0.2	0.4
Serum potassium (mEq/L)	4.1 ± 0.1	4.1 ± 0.1	1.0
CaxP (mg^2^/dL^2^)	31.9 ± 1.4	29.6 ± 1.9	0.3
Total serum proteins (g/dL)	7.5 ± 0.2	7.4 ± 0.1	0.8
CRP (mg/L)	2.9 ± 0.0	4.2 ± 1.3	0.3
Urinary creatinine (mg/dL)	103.2 ± 11.0	157.1 ± 29.4	0.1
Proteinuria (mg/dL)	89 ± 10.6	125.9 ± 17.9	0.1

Data are expressed as mean ± SEM, differences tested by Mann–-Whitney test. Abbreviations: BMI (body mass index), BP (blood pressure), HbA1c (glycated hemoglobin), HDL (high-density lipoprotein), LDL (low-density lipoprotein), CaxP (calcium-phosphorus product), CRP (C-reactive protein).

**Table 3:** Changes in clinical parameters after the treatment in the CKD group.

	Treatment	T0	T2	T3	Difference Between Time Points (*p*-Value)	Difference Between Arms (*p*-Value)
BMI	P	25.8 ± 0.9	26.0 ± 0.9	26.0 ± 0.9	-	-
	**S**	**27.2 ± 1.0**	**27.0 ± 1.1**	**27.4 ± 1.0**	-	
eGFR (mL/min)	P	23.9 ± 3.6	22.7 ± 3.0	23.3 ± 3.3	-	-
	**S**	**31.5 ± 2.8**	**31.1 ± 2.4**	**31.4 ± 2.5**	-	
Serum creatinine (mg/dL)	P	3.2 ± 0.4	3.1 ± 0.3	3.1 ± 0.3	-	*p* = 0.02
	**S**	**2.3 ± 0.2 $**	**2.3 ± 0.2 $**	**2.3 ± 0.2 $**	-	
Serum albumin (g/dL)	P	3.9 ± 0.1	3.9 ± 0.1	3.8 ± 0.1	-	-
	**S**	**3.9 ± 0.1**	**4.0 ± 0.1**	**4.0 ± 0.1**	-	
Azotemia (mg/dL)	P	97.0 ± 16.0	95.2 ± 16.4	97.5 ± 15.2	-	-
	**S**	**94.9 ± 7.2**	**94.1 ± 7.0**	**87.1 ± 6.2 §**	**0.01**	
Serum calcium (mg/dL)	P	9.2 ± 0.1	9.2 ± 0.2	9.0 ± 0.1	-	-
	**S**	**9.2 ± 0.1**	**9.2 ± 0.1**	**9.1 ± 0.2**	-	
Serum phosphorus (mg/dL)	P	3.4 ± 0.3	3.42 ± 0.2	3.2 ± 0.2	-	-
	**S**	**3.5 ± 0.1**	**3.4 ± 0.1**	**3.3 ± 0.1**	-	
Serum potassium (mEq/L)	P	4.7 ± 0.1	4.5 ± 0.1	4.5 ± 0.1	-	-
	**S**	**4.5 ± 0.1**	**4.5 ± 0.1**	**4.5 ± 0.1**	-	
Serum sodium (mEq/L)	P	141.0 ± 0.6	140.5 ± 0.5	138.4 ± 0.8 *§	*p* < 0.02	*p* = 0.001
	**S**	**142.1 ± 0.6**	**142.7 ± 0.4 $**	**140.7 ± 0.8**		
CaxP (mg^2^/dL^2^)	P	31.6 ± 2.7	31.6 ± 2.5	19.7 ± 4.2	-	-
	**S**	**32.3 ± 1.4**	**31.8 ± 1.1**	**23.7 ± 3.2 §**	**0.03**	
Total serum proteins (g/dL)	P	7.2 ± 0.1	7.2 ± 0.2	7.1 ± 0.1	-	-
	**S**	**7.2 ± 0.1**	**7.1 ± 0.1**	**7.1 ± 0.1**	-	
CRP (mg/L)	P	4.1 ± 1.1	3.1 ± 0.1	3.0 ± 0.1	-	-
	**S**	**3.0 ± 0.1**	**4.2 ± 1.2**	**3.3 ± 0.4**	-	
Urinary creatinine (mg/dL)	P	66.7 ± 9.5	59.4 ± 8.7	58.3 ± 7.6	-	-
	**S**	**53.7 ± 3.6**	**51.8 ± 4.5**	**48.9 ± 3.7**	-	
Urinary proteins (mg/dL)	P	577.0 ± 211.0	414.2 ± 139.6	431.8 ± 135.4	-	-
	**S**	**500.9 ± 117.9**	**497.8 ± 108.8**	**502.1 ± 116.3**	-	

Changes in clinical parameters after the treatment with either placebo or synbiotic (T2) and after the washout (T3) in the group of 23 CKD patients that completed the study. Normal character indicates mean results for arm P (*n* = 10), bold character indicates mean results for arm S (*n* = 13). * *p* < 0.05 vs. T0, § *p* < 0.05 vs. T2, $ *p* < 0.05 vs. P. Data are expressed as mean ± SEM, differences tested by Kruskal–Wallis multiple-comparison *z*-value test. Abbreviations: BMI (body mass index), eGFR (estimated glomerular filtration rate), CaxP (calcium-phosphorus product), CRP (C-reactive protein).

**Table 4:** Changes in clinical parameters after the treatment in the healthy group.

	Treatment	T0	T2	T3	Difference Between Time Points (*p*-Value)	Difference Between Arms (*p*-Value)
BMI	P	23.5 ± 0.8	23.3 ± 0.8	23.4 ± 0.8	-	-
	**S**	**26.0 ± 1.6**	**26.0 ± 1.6**	**26.3 ± 1.6**	-	
Serum creatinine (mg/dL)	P	0.79 ± 0.03	0.83 ± 0.04	0.77 ± 0.03	-	-
	**S**	**0.83 ± 0.05**	**0.90 ± 0.06**	**0.84 ± 0.05**	-	
Serum albumin (g/dL)	P	4.2 ± 0.1	4.1 ± 0.1	4.1 ± 0.1	-	-
	**S**	**4.1 ± 0.1**	**3.6 ± 0.4**	**4.0 ± 0.1**	-	
Azotemia (mg/dL)	P	32.6 ± 1.1	33.4 ± 2.1	31.2 ± 1.7	-	-
	**S**	**36.4 ± 3.1**	**33.3 ± 3.1**	**33.8 ± 3.0**	-	
Serum calcium (mg/dL)	P	9.4 ± 0.1	9.3 ± 0.2	7.1 ± 1.1	-	-
	**S**	**9.2 ± 0.1**	**9.1 ± 0.1**	**8.0 ± 1.0**	-	
Serum phosphorus (mg/dL)	P	3.4 ± 0.1	3.4 ± 0.2	3.4 ± 0.1	-	-
	**S**	**3.2 ± 0.2**	**3.3 ± 0.2**	**3.4 ± 0.2**	-	
Serum potassium (mEq/L)	P	4.1 ± 0.1	4.0 ± 0.1	4.1 ± 0.1	-	-
	**S**	**4.1 ± 0.1**	**4.0 ± 0.1**	**4.1 ± 0.1**	-	
CaxP (mg^2^/dL^2^)	P	31.9 ± 1.4	31.8 ± 1.5	23.8 ± 3.9	-	-
	**S**	**29.6 ± 1.9**	**29.9 ± 1.9**	**26.4 ± 3.9**	-	
Total serum proteins (g/dL)	P	7.5 ± 0.2	7.2 ± 0.1	7.3 ± 0.1		-
	**S**	**7.4 ± 0.1**	**6.9 ± 0.4**	**7.2 ± 0.1**	-	
CRP (mg/L)	P	2.90 ± 0.04	2.9 ± 0.04	2.9 ± 0.00	-	-
	**S**	**4.2 ± 1.3**	**3.5 ± 0.4**	**3.2 ± 0.2**	-	
Urinary creatinine (mg/dL)	P	103.2 ± 11.0	112.2 ± 13.7	101.1 ± 17.5	-	-
	**S**	**157.1 ± 29.4**	**154.0 ± 43.8**	**136.2 ± 20.5**	-	
Urinary proteins (mg/dL)	P	89.0 ± 10.6	85.1 ± 9.0	76.2 ± 8.1	-	-
	**S**	**125.9 ± 17.9**	**109.2 ± 23.8**	**103.2 ± 13.1**	-	

Changes in clinical parameters after the treatment with either placebo or synbiotic (T2) and after the washout (T3) in the group of 24 healthy volunteers that completed the study. Normal character indicates mean results for arm P (*n* = 14), bold character indicates mean results for arm S (*n* = 10). Data are expressed as mean ± SEM, differences tested by Kruskal–Wallis multiple-comparison *z*-value test. Abbreviations: BMI (body mass index), CaxP (calcium-phosphorus product), CRP (C-reactive protein).

**Table 5:** Baseline whole gastro-intestinal permeability in healthy subjects and in CKD patients.

	Healthy Subjects	CKD III B	CKD IV	*p*
Number	27	11	11	
Stomach (sucrose recovery, %)	0.031 ± 0.006	0.019 ± 0.007	0.021 ± 0.007	ns
Median	0.02	0.02	0.02	
Range	0.01–0.16	0.01–0.03	0.01–0.05	
Small intestine (lactulose/mannitol, ratio)	0.029 ± 0.01	0.019 ± 0.01	0.03 ± 0.01	ns
Median	0.017	0.018	0.017	
Range	0.005–0.31	0.012–0.032	0.01–0.1	
Colon (sucralose recovery, %)	1.03 ± 0.08	0.40 ± 0.09 *	0.30 ± 0.09 *	<0.00001
Median	0.97	0.4	0.31	
Range	0.01–1.95	0.25–0.65	0.16–0.51	

Data are expressed as mean ± SEM, median and range. Differences tested by one-way ANOVA followed by Fisher’s LSD Multiple comparison test. Significance levels: * *p* < 0.05 vs. healthy subjects.

**Table 6:** Effect of chronic placebo or synbiotic administration on gastrointestinal permeability in CKD patients.

**Placebo**	**Baseline**	**T2**	**T3**	***p*-Value**
Number	9	9	9	
Stomach (sucrose recovery, %)	0.02 ± 0.003	0.018 ± 0.003	0.021 ± 0.003	ns
Median	0.02	0.02	0.02	
Range	0.01–0.05	0.01–0.03	0.01–0.04	
Small intestine (lactulose/mannitol, ratio)	0.027 ± 0.006	0.022 ± 0.006	0.02 ± 0.006	ns
Median	0.018	0.02	0.02	
Range	0.012–0.10	0.014–0.035	0.01–0.035	
Colon (sucralose recovery, %)	0.36 ± 0.06	0.37 ± 0.07	0.34 ± 0.06	ns
Median	0.32	0.37	0.36	
Range	0.16–0.56	0–0.08	0.14–0.57	
**Synbiotic**	**Baseline**	**T2**	**T3**	***p*-Value**
Number	12	12	12	
Stomach (sucrose recovery, %)	0.017 ± 0.002	0.017 ± 0.002	0.015 ± 0.002	ns
Median	0.02	0.015	0.01	
Range	0.01–0.03	0.01–0.03	0.01–0.03	
Small intestine (lactulose/mannitol, ratio)	0.019 ± 0.01	0.015 ± 0.001 *	0.015 ± 0.001 *	0.040
Median	0.017	0.015	0.014	
Range	0.01–0.032	0.008–0.022	0.01–0.023	
Colon (sucralose recovery, %)	0.35 ± 0.04	0.41 ± 0.05	0.36 ± 0.05	ns
Median	0.34	0.4	0.31	
Range	0.2–0.65	0.2–0.7	0–0.82	

Data expressed as mean ± SEM, median and range. Differences tested by one-way ANOVA followed by Fisher’s LSD Multiple comparison test. * *p* < 0.05 vs. baseline.

**Table 7:** Effect of placebo or synbiotic administration on gastrointestinal permeability in healthy subjects.

**Placebo**	**Baseline**	**T2**	**T3**	***p*-Value**
Number	14	14	14	
Stomach (sucrose recovery, %)	0.027 ± 0.004	0.028 ± 0.004	0.025 ± 0.002	ns
Median	0.02	0.025	0.03	
Range	0.01–0.06	0.01–0.06	0.01–0.04	
Small intestine (lactulose/mannitol, ratio)	0.018 ± 0.003	0.18 ± 0.002	0.016 ± 0.001	ns
Median	0.016	0.016	0.017	
Range	0.009–0.047	0.01–0.05	0.009–0.025	
Colon (sucralose recovery, %)	1.03 ± 0.07	1.14 ± 0.09	1.04 ± 0.1	ns
Median	1.04	1.12	0.98	
Range	0.64–1.45	0.63–1.64	0.58–2.15	
**Synbiotic**	**Baseline**	**T2**	**T3**	***p*-Value**
Number	10	10	10	
Stomach (sucrose recovery, %)	0.037 ± 0.012	0.035 ± 0.007	0.045 ± 0.016	ns
Median	0.02	0.02	0.02	
Range	0.01–0.16	0.02–0.07	0.01–0.17	
Small intestine (lactulose/mannitol, ratio)	0.042 ± 0.025	0.015 ± 0.002	0.017 ± 0.002	ns
Median	0.018	0.015	0.015	
Range	0.005–0.31	0–0.022	0.011–0.031	
Colon (sucralose recovery, %)	1.04 ± 0.17	0.98 ± 0.07	1.13 ± 0.13	ns
Median	0.94	0.92	1.17	
Range	0.01–1.95	0.76–1.44	0.45–1.68	

Data expressed as mean ± SEM, median and range. Differences tested by one-way ANOVA followed by Fisher’s LSD Multiple comparison test.

**Table 8:** GSRS scores in the CKD group (13 S and 10 P).

		T0	T2	T3	*p*-Value Between Time Points
GSRS 6	P	2.40 ± 0.41	2.20 ± 0.51	2.00 ± 0.32	-
	S	2.38 ± 0.34	2.08 ± 0.41	1.46 ± 0.23 *	0.04
GSRS 13	P	2.20 ± 0.76	1.40 ± 0.38	1.40 ± 0.38	-
	S	2.23 ± 0.27	1.15 ± 0.15 *	1.31 ± 0.20 *	<0.004
Abdominal pain	P	4.00 ± 0.58	4.40 ± 0.75	3.40 ± 0.25	-
	S	4.85 ± 0.51	3.54 ± 0.28 *	3.62 ± 0.34	0.03 *
Constipation syndrome	P	5.00 ± 0.69	4.00 ± 0.58	4.40 ± 0.64	-
	S	6.08 ± 0.80	3.77 ± 0.41*	4.23 ± 0.71	0.02 *

Only GSRS items and domains showing statistically significant changes after the treatment (items 4, 6 and 13, abdominal pain, indigestion syndrome and constipation syndrome) are shown. * *p* < 0.05 vs. T0. Data are expressed as mean ± SEM, differences tested by Kruskal–Wallis multiple-comparison *z*-value test.

**Table 9:** GSRS scores in the healthy volunteer group (10 S and 14 P).

		T0	T2	T3	*p*-Value Between Time Points
GSRS 3	P	2.08 ± 0.28	1.15 ± 0.15 *	1.15 ± 0.15	0.01
	S	1.67 ± 0.32	1.00 ± 0.00	1.44 ± 0.28	-
GSRS 9	P	3.15 ± 0.26	1.77 ± 0.27 *	2.08 ± 0.35	0.01
	S	2.33 ± 0.32	3.00 ± 0.55	2.11 ± 0.46	-
Abdominal pain	P	8.69 ± 0.81	5.92 ± 0.42 *	6.23 ± 0.51 *	<0.01
	S	7.33 ± 0.98	5.33 ± 0.22	6.56 ± 1.26	-
Indigestion syndrome	P	9.69 ± 0.95	6.31 ± 0.53 *	6.46 ± 0.66 *	<0.01
	S	7.56 ± 0.99	8.44 ± 1.17	6.89 ± 1.15	-

Only GSRS items and domains showing statistically significant changes after the treatment (items 3, 9 and 15, abdominal pain, indigestion syndrome) are shown. * *p* < 0.05 vs. T0. Data are expressed as mean ± SEM, differences tested by Kruskal–Wallis multiple-comparison *z*-value test.

## Data Availability

The data presented in this study are available on request from the corresponding author.

## Supplementary Materials

The following are available online at https://www.mdpi.com/article/10.3390/toxins13050334/s1, Figure S1: Serum levels of IL-6 concentrations in CKD patients before and after the treatment with the synbiotic.
