# Supplementary material for: An Innovative Synbiotic Formulation Decreases Free Serum Indoxyl Sulfate, Small Intestine Permeability and Ameliorates Gastrointestinal Symptoms in a Randomized Pilot Trial in Stage IIIb-IV CKD Patients
Source: Toxins (Basel). 2021 May 5;13(5):334. doi: 10.3390/toxins13050334 (PMC8147955; doi:10.3390/toxins13050334)
Supplement: Supplementary file 1 [file toxins-13-00334-s001.zip › toxins-1176737-supplementary.pdf]

Supplementary Materials: An Innovative Synbiotic Formulation Decreases Free Serum Indoxyl Sulfate, Small Intestine Permeability and Ameliorates Gastrointestinal Symptoms in a Randomized Pilot Trial in Stage IIIb-IV CKD Patients

Carmela Cosola, Maria Teresa Rocchetti, Ighli di Bari, Paola Maria Acquaviva, Valentina Maranzano, Simone Corciulo, Agostino Di Ciaula, Domenica Maria Di Palo, Flavia Maria La Forgia, Sergio Fontana, Maria De Angelis, Piero Portincasa and Loreto Gesualdo

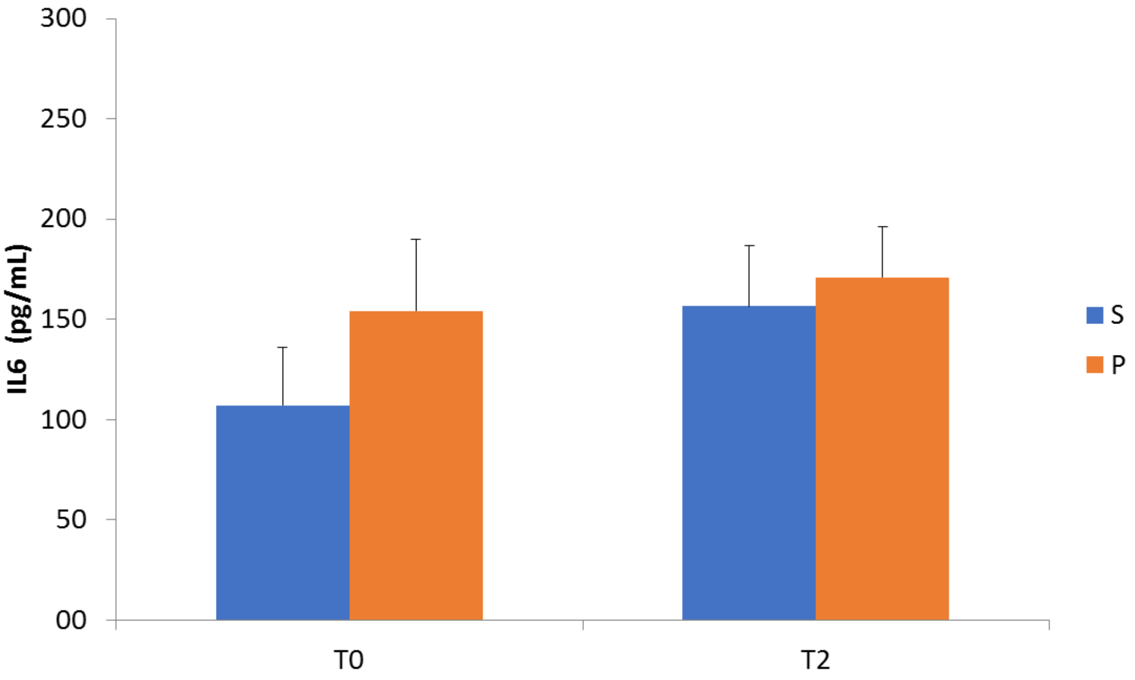

Figure S1. Serum levels of IL-6 concentrations in CKD patients before and after the treatment with the synbiotic.
